# Pros and cons of CLA consumption: an insight from clinical evidences

**DOI:** 10.1186/1743-7075-12-4

**Published:** 2015-02-03

**Authors:** Sailas Benjamin, Priji Prakasan, Sajith Sreedharan, Andre-Denis G Wright, Friedrich Spener

**Affiliations:** Biotechnology Division, Department of Botany, Enzyme Technology Laboratory, University of Calicut, Kerala, 673 635 India; School of Animal and Comparative Biomedical Sciences, University of Arizona, Tucson, AZ 85721 USA; Department of Molecular Biosciences, University of Graz, Heinrichstrasse 31, 8010 Graz, Austria

**Keywords:** Conjugated linoleic acids, CLA, Review, Clinical evidences

## Abstract

This comprehensive review critically evaluates whether supposed health benefits propounded upon human consumption of conjugated linoleic acids (CLAs) are clinically proven or not. With a general introduction on the chemistry of CLA, major clinical evidences pertaining to intervention strategies, body composition, cardio-vascular health, immunity, asthma, cancer and diabetes are evaluated. Supposed adverse effects such as oxidative stress, insulin resistance, irritation of intestinal tract and milk fat depression are also examined. It seems that no consistent result was observed even in similar studies conducted at different laboratories, this may be due to variations in age, gender, racial and geographical disparities, coupled with type and dose of CLA supplemented. Thus, supposed promising results reported in mechanistic and pre-clinical studies cannot be extrapolated with humans, mainly due to the lack of inconsistency in analyses, prolonged intervention studies, follow-up studies and international co-ordination of concerted studies. Briefly, clinical evidences accumulated thus far show that CLA is not eliciting significantly promising and consistent health effects so as to uphold it as neither a functional nor a medical food.

## Introduction

**Figure 1:**
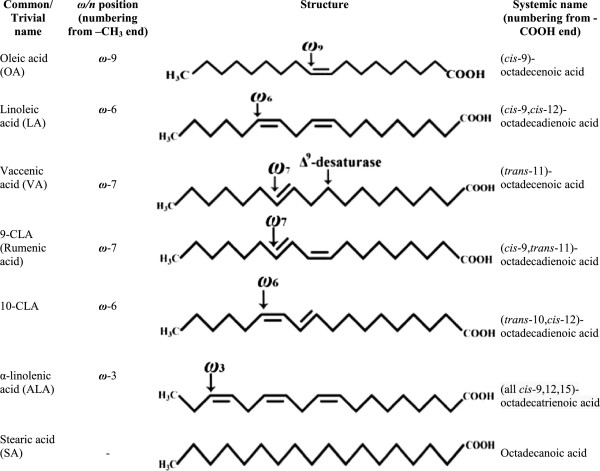
**Major** ***ω*** **fatty acids with their common names, structures and systemic names.**

Conjugated linoleic acids (CLAs) encompass a group of positional and geometric isomers of octadecadienoic acids (18:2) – naturally occurring polyunsaturated fatty acids or PUFA- synthesized in the rumen of cattle, deer, sheep and goat by microbial biotransformation of forage-derived fatty acids (FAs) such as oleic acid (OA), linoleic acid (LA) and *α*-linolenic acid (ALA) ultimately into saturated stearic acid (SA) [1, 2]. Although CLA is formed as an intermediate during ruminal biohydrogenation of OA, LA and ALA, its primary source *in vivo* is endogenous (*de novo*) synthesis by the activity of Δ^9^-desaturase from the monounsaturated FA (MUFA), the vaccenic acid (*trans*-11,18:1; VA), another intermediate in ruminal biohydrogenation [3]. It is also synthesized endogenously in humans from dietary VA by the activity of Δ^9^-desaturase [4, 5] (Figure 1). The Δ^9^-desaturase (also referred to as stearoyl-CoA desaturase; EC 1.14.99.5) catalyzes the addition of a *cis*-9 double bond on the VA, and was shown to be present in several tissues, including the mammary gland, adipose, liver, and intestine; during the process, *cis*-9, *trans*-11-CLA (designated as 9-CLA, the rumenic acid) is formed from VA [6]. Thus, VA is the pivotal precursor of 9-CLA in ruminants (probably in mammals too); therefore, an essential FA in humans.

In fact, most of the commercially available CLAs are produced by the alkaline isomerization of LA-rich oils, such as sunflower oil, and tend to contain an equimolar mixture of 9- and 10-CLAs, together with a mixture of variable quantities (up to 30%) of other geometrical and positional isomers of CLA, and that 100% pure CLA isomer is not available on the market [7]. Therefore, the primary focus of all mechanistic, preclinical and clinical studies pertaining to CLA were on 9- and 10-CLAs – especially a mixture of both or rarely one of these two isomers (with others isomers as impurities) [8, 9]. During the past couple of decades, hundreds of reports - principally based on *in vitro*, microbial, animal, and of late clinical studies on humans - have been accumulating with the highlights of contrasting biological activities of CLA isomers, especially of 9- and 10-CLAs [9, 10].

There has been an overwhelming consumer interest toward the health improving role of specific foods or food components, the so-called ‘functional foods’ [8]. The term ‘functional food’ is often used as a generic description for the beneficial health effects of ingested foods that go beyond their traditional nutritive values. The supposed health benefit of CLA was discovered nearly three decades ago, *i.e.*, Ha and his co-workers found that ground beef contained an anti-carcinogenic factor that consisted of a series of conjugated dienoic isomers of LA [11]. As the biomedical studies with CLA expanded, it became apparent that CLA showed a range of positive health effects in experimental animal models. Such supposed beneficial health effects were attributed to suppressing cancer, reducing body fat accretion, delaying the onset of type II diabetes, retarding the development of atherosclerosis, improving the mineralization of bone and modulating the immune system [12–14]. Therefore, CLA-rich foods may be considered as functional foods (a food offers an additional function in the form of health-promotion or disease prevention in combination with some supporting ingredients); and that CLAs *per se* are neither a food nor a functional food but a FA class with some bioactive properties.

In the light of the aforesaid background, this review critically examines whether the health benefits attributed to CLA on humans are clinically proven or not. Keeping this in mind, this review is categorized into different sections with appropriate illustrations wherever necessary. The thrust areas addressed include: structure of CLA, intervention strategies, physiological effects of CLA consumption associated with diseases such as obesity, cardiovascular disorders, diabetes, cancer, immune disorders; certain adverse effects of CLA consumption such as oxidative stress, abdominal irritations, milk fat depression, insulin resistance, coupled with possible drawbacks such as ignoring the ingredients in placebo; differences in the duration of study, dosage of CLA, food/life styles, and selection of subjects.

### Structure of CLA

Very often, the CLAs are erroneously classified as *omega*-6 (abbreviated as *ω*-6 or *n*-6) FAs. In fact, CLA is a class of FAs comprising as many as 56 isomers with conjugated (juxtaposed or neighboring) double bond pairs (*i.e*., at positions 6,8-; 7,9-; 8,10-; 9,11-; 10,12-; 11,13-; 12,14-; and 13,15- with *cis-cis, cis-trans, trans-cis* or *trans-trans* geometric configurations) varying along octadecadienoic acid (18:2) [15] (Figure 1). Regarding geometric isomers, the *cis* and *trans* configurations unequivocally indicate the steric relations around a (given) double bond; however, instead of *cis* and *trans,* the symbols *Z* (from German *zusammen,* means together) and *E* (from German *entgegen*, means opposite), respectively are used in some classifications. It should not be confused with the counting of carbon position from *ω* (-CH_3_) or –COOH terminus along the acyl chain; the former is used in *ω* classification, while the latter in systemic nomenclature. From this, it is evident that only the isomers with the conjugated double bonds on carbon positions starting at 10 (from –COOH terminus) have indeed their first C-C double bond at the *ω*-6 position, while counting from the methyl (-CH_3_) terminus (*i.e*. from the last or *omega*-C in the acyl chain) (Figure 1). Thus, *trans*-10, *cis*-12-CLA (designated as 10-CLA) is a typical *ω*-6 CLA; while the biologically active and predominantly (natural) occurring 9*-*CLA is a typical *ω*-7 FA [8]. Though, VA (the principal precursor of 9-CLA) is devoid of conjugated double bonds; it is also an *ω*-6 FA, while counting from the methyl terminus on the acyl chain (Figure 1).

### Intervention strategies

Clinical studies are generally of two categories: the cross-over and non cross-over (parallel or between patients) designs; in the former, the subjects are randomized into two groups of which the first receive X (*e.g*., a drug) followed by Y (*e.g*., a placebo), and the second group receive Y first, followed by X; while in the non cross-over category also two groups will be there – one of the groups (the test group) will invariably receive X and the other group (the control group) receive Y, then the results are compared between the test and control groups (*i.e*., parallel). In cross-over study, the influence of covariates is reduced as each cross-over patient serves as her or his own control; while in non cross-over study, the treatment groups may be unbalanced on certain covariates [16]. Unlike non cross-over study, cross-over studies are statistically efficient, and hence fewer subjects are required for the study. In fact, in non cross-over design, the placebo effect will not be mixed up with the effect of the test material. Thus, both methods have some advantages and disadvantages in different situations, and hence, selection of study design depends on the situation of the study.

Generally, the clinical studies on the efficacy of CLA are randomized, double-blind and placebo-controlled designs comprising two groups, *i.e*., one group of subjects (the experimental group) receive the active CLA isomers (*i.e*., 9- or 10-CLA) or its mixture; and the other half (control group) receive a placebo, designed without CLA or its isomeric mixture. In such experiments, neither the researcher nor the subjects know whether they received CLA or the placebo designed for the purpose (*i.e*., they are “blind”) till all the data are recorded. Such types of clinical studies ensure that the personal expectations of neither the researcher nor the subjects influence the results, making it more dependable, and thus eliminate possible treatment bias. Nevertheless, in case of some life-threatening diseases like cancer, analyses of the implications on the direct supplementation of CLA seem to be difficult. In such cases, epidemiological studies were performed in which intake data derived from a validated food-frequency questionnaire are linked to an existing or freshly established nutrient databases containing analytical data of specific FA [16–18]. These studies may be limited by the variability of CLA in the food supply as well as the difficulty of assessing the intake of these minor dietary FAs, the CLA. Some of the commercially available CLA preparations, commonly used for clinical studies are detailed in Table 1.

**Table 1:** **Commercially available common CLA mixtures with their FA composition, trade name and manufacturer**

Trade name	CLA content (%)	Total CLA (%)	Other FAs (%)*	Physical form	Manufacturer	Reference
Clarinol™	9*-*CLA (37.3);	79.4	20.6	capsule	Loders Croklaan,	[19]
	10*-*CLA (37.6);				The Netherlands	
	other CLAs (4.5)					
CLA-80	9-CLA (41.6);	82	18	capsule	Cognis Corporation,	[20]
	10-CLA (40.4)				The USA	
Tonalin-TG	9-CLA (37.49);	80.8	19.2	capsule	Natural lipids,	[21]
	10-CLA (38.02);				Norway	
	other CLAs (5.26)					
Tonalin™	9-CLA (39.2);	79.6	20.4	capsule	Cognis	[22]
	10-CLA (38.5);				Corporation, The USA	
	other CLAs (1.9)					
Tonalin®	9-CLA (11.4);	65	35	capsule	Pharmanutrients, Inc.	[23]
	10-CLA (14.7);				Lake Bluff, IL	
	other CLAs (38.9)					
CLA-Capsules	9-CLA (21.7) ;	56.6	43.4	capsule	Fitness Pharma, Norway	[21]
	10-CLA (19.1);					
	other CLAs (15.8)					
CLA-enriched Margarines and yoghurts	9-CLA (14.6);	19.3		drink	NIZO Food Research,	[24]
	10-CLA (3.3)				The Netherlands	
	other CLAs (1.4)					
CLA-70	9-CLA (33.8);	69	31	capsule	TrofoCell, Hamburg,	[25]
	10-CLA (35.2)				Germany	

*includes FAs such as OA, LA, ALA, SA, palmitic acid, *etc.*in varying concentrations.

Most of the clinical studies monitored the effect of commercially available CLA supplements, which usually contain a mixture of 9- and 10-CLAs at approximately 50:50 ratio, whereas some other researchers used naturally CLA-enriched dairy products for evaluating the biological activities. Typical level of CLA in cow’s milk fat is about 0.5% [26, 27]; which would vary considerably depending on the composition of diet [*e.g*., green forage, organic forage, nature and age of forage (young leaves of grasses better), altitude of grazing, silage and concentrates supplemented to the feed for fortification], coupled with stage of lactation, lactation number, breed, parity, animal’s health, climate, ruminal micobiota (especially bacteria and protozoa), *etc.*[26–30].Feeding animals with plant oils rich in LA or ALA (such as sunflower, soybean or linseed oil) is shown to enhance the 9-CLA content in the milk fat, which can subsequently be used to make CLA-enriched dairy products [31], *i.e*., an *in situ* approach [32]. For instance, the CLA content could be enhanced up to 2.08% of total milk FAs, if 4% (in terms of the dry weight of feed) soybean oil (is supplemented as concentrate in the feed) [33]; as reported in the case, wherein flaxseed or fish oil supplemented to the diet [34]. Naturally CLA-enriched (*in situ*) dairy products such as butter, cheese, *etc*. were incorporated in a variety of recipes such as those used for making muffins, cakes, sauces, and very often used as spreads.

### CLA and body composition

Overweight or obesity - one of the typical syndromes of lifestyle diseases - is referred to as the excessive fat accumulation that impacts health. The occurrence of overweight and obesity has been augmented as the most common health issue of modern food style. Obesity is considered as a cause for many health problems such as heart diseases, infertility and insulin resistance [35]. With a view to lose weight and improve the body composition (*i.e*., increase in fat-free mass and decrease in body fat, many diet supplements, health preparations/formulations or weight-loss drugs were made available on the market by various companies. Among them, CLAs draw more attention, since many pre-clinical studies in animal models proved its inverse relationship with obesity. The significant clinical studies investigating the effect of CLA on body composition and their intervention strategies are shown in Table 2.

**Table 2:** **List of clinical trials investigating the effect of CLA consumption on body composition; ↑- increased; ↓- decreased; ↔ no change in**

Subjects	Body type	Age	BMI kg/m ^2^	Dosage (g/d)	Composition	Placebo	Duration	Measurement	Effect of CLA	Country/State	Reference
60	Overweight/obese			1.7, 3.4, 5.1 or 6.8	9 and 10-CLA (50:50)	Olive oil	12 wk	Dual-energy X-ray absorptiometry	↓ bodyfat mass	Norway	[36]
54	Overweight	20-50	27.81 ± 1.5	1.8 or 3.6	Tonalin™	OA	13 wk	Hydrodensitometry/deuterium dilution	↑ body weight maintenance after weight loss	The Netherlands	[37]
17	Normal	20-41	-	3	CLA Pharmanutrients Inc.	Sunflower oil	64 d	Dual x-ray absorptiometry (DXA)	↔ body weight	The USA	[38]
60	Overweight/obese	>18	27.5-39.0	3.4	Tonalin™	Olive oil	12 wk	bioelectrical impedance, Dual x-ray absorptiometry	↓ Mean body weight & BMI	Norway	[39]
48	Normal/obese	18-50	30-35	3.2 or 6.4	9 & 10-CLA (50:50)	Safflower oil	12 wk	Dual-energy X-ray absorptiometry	↑ LBM	The USA	[40]
24	Normal	19–24	>30	0.7-1.4	9 & 10-CLA (50:50)	Soybean oil	8 wk	Skinfold thickness	↓ bodyfat mass	-	[25]
20	Normal	18-30	>25	1.8	Tonalin™	Hydrogel	12 wk	Near infrared light utilizing a Futrex 5000 A instrument	↓ body fat ↔ body weight	Norway	[41]
23	Normal			3	Tonalin™	Olive oil	28 d	Dual-energy X-ray absorptiometry	↔ fat mass	The USA	[42]
30	Overweight/obese	35-55	>25	3.2	Tonalin™	Safflower oil	12 wk	Computed tomography	↔ visceral adipose tissue	The USA	[22]
118	Overweight/obese	18-65	28–32	3.4	Clarinol™	Olive oil	6 mnt	Waist–hip ratio	↓ bodyfat mass	Norway	[43]
85	Overweight/obese	45-68	25–35	4.5	Tonalin TG 80	Safflower/olive oil	4 wk	Waist/hip ratio	↓ body weight	Germany	[44]
32	Normal			1.3	naturally or synthetically with 9 & 10-CLA	Untreated milk	8 wk	Magnetic resonance imaging	↔ fat mass	Canada	[45]
81	overweight	35-65	25-30	1.5/3	Dairy drink with 9 & 10-CLA	OA/ sunflower oil	18 wk	Dual-energy X-ray absorptiometry	↔ body composition	The Netherlands	[46]
60	overweight	35-65	25–35	3	Milk with 9 & 10-CLA	Skimmed milk	12 wk	Dual-energy X-ray absorptiometry	↓ body fat mass	Spain	[47]
55	Obese	≤70	>30	8	CLA mixture	Safflower oil	36 wk	Anthropometry	↔ BMI; ↑ lean mass	The USA	[20]
76	Normal	18 - 45	-	5	Tonalin	Sunflower oil	14 wk	Air displacement plethysmography	↑ lean tissue mas;↓ body fat mass	Canada	[48]
81	Postmenopausal women	>35		4.7	9 & 10-CLA (50:50)	Olive oil	8 wk	Dual energy X-ray absorptiometry	↓ total fat mass and lower body fat mass	Denmark	[49]
33	Type-2 diabetes patients	35-50	25-30	8	9 & 10-CLA (50:50)	Soybean oil	8 wk	Bioelectrical impedance, anthropometry	↔ body composition	Iran	[50]

In clinical studies, several methods or techniques were applied to measure the body composition. The simplest method is that, to measure the thickness of subcutaneous fat in multiple places on the body such as abdominal area, arms, sub-scapular region (large triangular muscle near shoulder bone), buttocks and thighs [25, 51, 52]. Other commonly used measures are bioelectrical impedance analysis [39], hydrodensitometry and dual-energy X-ray absorptiometry (DEXA) [53–55]. Among them, DEXA is the most widely used method in clinical studies to assess body composition due to CLA consumption. In fact, DEXA measures total body composition and fat content with a high degree of accuracy and is considered as the gold standard for measuring the body composition, since one can get an image of the entire body [56]. Another commonly used method is the measurement of body mass index, *i.e*., a measure of body weight to height index of a person which is calculated as weight in kilograms divided by the square of height in meters (kg/m^2^). According to World Health Organization [57], a BMI greater than or equal to 25 is overweight, and a BMI greater than or equal to 30 is considered as obesity. In fact, BMI is not necessarily a good measure, especially in terms of body composition; for instance, individuals like athletes with strong bone and greater muscle (lean) mass have a higher BMI than non-athletes, and hence different BMI classifications of overweight is warranted while assessing obesity in terms of body girth [58].

Some of the clinical studies suggested a positive association of the intake of 3.4 to 6.8 g/d isomeric mixture of CLA (mainly 9- and 10-CLA) supplementation for 12 wk for overweight and obese volunteers (BMI, 25 to 35 kg/m^2^) of either sex in reducing the body fat mass (BFM) significantly [36]. In another study, supplementation of 4.2 g/d isomeric mixture of 9- and 10-CLAs for 4 wk decreased the sagittal abdominal diameter in obese individuals; but, body weight and BMI remained unaffected [59, 60]. In a different study comprising 60 overweight or obese volunteers including men and women who received 3.4 g/d CLA for 12 wk showed reduction in the mean weight and mean BMI, and these results indicated that CLA in the given dose was safe in healthy populations with regard to the safety parameters investigated [39]. Steck et al. [40] examined the effect of 2 doses of CLA (3.2 g/d or 6.4 g/d) for 12 wk (mixture of 9- and 10 CLA in 50:50 ratio) on body composition in obese individuals, who were free of chronic diseases. They concluded that lean body mass (LBM) increased by the higher dose after 12 wk of intervention. Supplementation of 9- and 10-CLAs at a dosage of 1.7 g/d for 12 wk in overweight and class I (low risk) obese subjects (*i.e*., BMI = 30.0 – 34.9) of Chinese population showed lower obesity indices with no obvious adverse effects [61]. Interestingly, CLA was found to be effective in reducing the weight gain associated with psychiatric medications - one of the major side effects in psychological treatments. Consumption of CLA at a dosage of 3.4 g/d along with green tea extract significantly reduced total body fat percentage in psychiatric patients by 5.1 to 8.1% and increased LBM by 4.4 to 11% [62]. A possible explanation for this effect is that green tea extract high in pigallocatechin-gallate can directly inhibit gastric and pancreatic lipases, thereby increasing the thermogenesis and possibly prevent the enzymatic degradation of catechol O-methyltransferase, an enzyme which plays a role in the respiration rate of brown adipose tissue [62].

Due to overeating and sedentary life, the incidence of weight gain during holiday season (*i.e.,* obesity and overweight) has increased considerably during the past two decades and currently affects majority of the adult population. For instance, supplementation of CLA (3.2 g/d for 6 month) for 40 healthy overweight adults (18–44 yr; BMI: 25–30 kg/m^2^), significantly reduced body fat (by 1.0-2.2 kg) and prevented weight gain during their holiday season [63]. Thus, all these studies suggest some potential beneficial effects upon consumption of isomeric CLA mixture (2 to 6 g/d) in body composition of obese or overweight individuals.

On the contrary, an inverse relationship between CLA and body composition has been demonstrated. One of the first studies demonstrating negative effects of CLA was performed with 71 subjects including obese men and women of 20 to 50 yr of age. The subjects were instructed to take 90% pure CLA (2.7 g/d active 9- and 10-CLA isomers in equal ratio) daily for 26 wk, and compared the effects to 3 g/d safflower oil as placebo. Body was measured by hydrodensitometry, but the results did not show any effect on body composition [64]. In sedentary young women, intake of 2.1 g CLA/d for 45 d did not induce any changes in body composition [52]. Likewise, consumption of 4.5 g/d CLA isomeric mixture showed no decrease in body weight, in comparison to the consumption of safflower oil as placebo in 85 overweight and obese male subjects [44].

Some of the studies observed gender specific effects of CLA intake. Riserus et al. [65] showed that CLA (4.2 g/d) supplementation for 4 wk in 14 obese men (BMI: 32 ± 2.7 kg/m^2^; 39 – 64 yr old) with the metabolic syndrome may decrease abdominal fat, without concomitant effects on overall obesity or other cardiovascular risk factors. The small sample size and short duration were the major limitations of this study; thus, the effects of CLA in abdominal obesity need to be investigated further in larger studies with longer duration. Long-term (1 yr) supplementation (daily dose of CLA was 3.6 g; the mixture contained 39% 9-CLA, 41% 10-CLA) with CLA in free FA or triacylglycerol (TAG) did not show significant improvement on the BFM in healthy overweight adults (higher standard deviation found in the reported value makes the report neutral) [66]. In this double-blind placebo-controlled study, 180 (female 149 and male 31) volunteers with BMI of 25–30 were included. In another study, healthy adult women were examined for the effects of an intake of 3 g/d CLA for 64 d on body composition, but no differences were found in the measured parameters like fat-free mass, BFM and percentage of BFM, body composition, energy expenditure, fat oxidation and respiratory exchange ratio against sunflower oil as placebo [38]. In a bicentric study (conducted simultaneously at Clermont-Ferrand, France and Maastricht, The Netherlands), eighty-one middle-aged, overweight, healthy men and women were enrolled, and all subjects consumed a drinkable dairy product containing 3 g of high OA sunflower oil daily (for 6 wk, the run-in period) [46].Volunteers were then randomized over five groups receiving daily either 3 g of high OA sunflower oil, 1.5 or 3 g each of 9- or 10-CLA administrated as TAG in a drinkable dairy product for 18 wk. Percentage BFM, fat and LBM were assessed at the end of the run-in and experimental periods by DEXA. Dietary intake was also recorded. It was concluded that, a daily consumption of a drinkable dairy product containing up to 3 g of CLA isomers for 18 wk had no significant effect on body composition in overweight, middle-aged men and women [46].

A study from Greece reported that CLA administered first at 0.7 g/d for 4 wk and thereafter at 1.4 g/d for the next 4 wk, decreased BFM in healthy volunteers [25]. Raff et al. [49] compared the effects of the supplementation of 5.5 g/d CLA mixture (50:50 mixture of 9- and 10-CLA) or only 9-CLA for 16 wk and assessed the change in total and regional fat mass in healthy postmenopausal women and concluded that the consumption of 9- and 10-CLA mixture resulted in the reduction of total and lower BFMs. Likewise, supplementation of 3 g/d of 80% CLA (50:50 ratio of 9- and 10-CLA) for 7 month attenuated the increase in LBM by 0.5 ± 0.8 (SD is on higher side). This study gained more importance, as it was reported in children aged between 6 and 10 yr, who were overweight or obese, but otherwise healthy [67]**]**. Interestingly, in a study to examine improvement on the reduction of BFM, CLA (500 mg/d) was supplemented in conjunction with 50 mg γ-oryzanol, which effectively reduced BFM by 1.14 kg (against 0.36 kg reduction in CLA group) in healthy overweight Korean women (n = 51, BMI > 23) [68]. It is known that the γ -oryzanol is a phytochemical having several biological activities like anti-oxidant activity, anti-atherogenic effect, lowering triglycerides and improves LBM [68]. This report also indicates that CLA *per se* was less efficient to improve BFM; for instance, a recent non cross-over clinical study conducted on 66 non-trained healthy male students for 2 month showed that CLA supplementation had no effect on LBM, BFM, trunk and visceral fats, and waist circumference [69].

From the aforesaid reports, it seems that a minimum daily dose (about 3 g/human) is required to induce reduction in fat. Some clinical studies suggested that administration of CLA might be the most effective strategy in controlling regionalized reduction of fat mass rather than its constitutional reduction, *i.e*., uniformly in the whole body. For instance, administration of 3.4 g/d CLA for 6 month reduced fat mass significantly in legs [43]. Waist-to-hip ratio also decreased significantly in healthy, overweight and obese men, compared with placebo group. Interestingly, these effects were produced independent of diet and specific lifestyle.

### Effect of CLA on exercise

Exercising individuals often add nutritional supplements to their diet to accelerate the increase in muscle mass and strength from heavy resistance-exercise training. Some short- and long-term studies employing high doses of CLA in healthy and obese, sedentary and exercised adults have shown beneficial effects of CLA in reducing fat mass and increasing LBM. A daily supplementation of 1.8 mg CLA for 12 wk reduced body fat (measured using near infrared lights); but not body weight in healthy exercising humans of normal body weight, compared to the placebo group who received hydrogel [41]. In this study, physical exercise was standardized as 90 min in gym, three times a wk; and concluded that CLA reduces the deposition of fat. These results seem to be encouraging, because much lower dose of CLA produced the expected results - when compared to other studies - wherein comparatively 2 to 4 folds higher concentrations of CLA were used. Effect of CLA (Clarinol A-80) supplementation in conjunction with 6 wk of aerobic exercise training on 33 untrained to moderately trained men (average age 21.6 ± 2.8 yr) was investigated [70]. CLA showed no ergogenic benefits on neuromuscular fatigue, and field tests of muscular endurance and power.

CLA also gained attention among resistance-trained athletes as agents for reducing catabolism, body fat and improving muscle mass during training, but supplementation of 6 g/d of CLA coupled with 3 g/d of FAs in the formulation (Tonalin®) against 9 g/d olive oil placebo for 28 d showed no significant ergogenic value, as it did not significantly affect changes in total body mass, fat-free mass, fat mass, percent body fat, bone mass, strength, serum substrates, or general markers of catabolism during training [42]. But in contrast, administration of 6 g/d CLA (in combination with 5 g/d creatine monohydrate) followed by resistance exercise training in older adults (above 65 yr, comprising 19 men and 20 women) for 6 month enhanced strength gains and improved body composition [71]. This combined strategy showed that supervised resistance exercise training is safe and effective for increasing strength in older adults, because aging is associated with lower muscle mass and an increase in body fat. In another study, CLA (6 g/d) supplementation along with creatine (9 g/d) and whey protein (36 g/d) also found beneficial for improving body strength and LBM during heavy resistance training in well-trained young adults (both men and women; aged 22.5 ± 2.5 yr) with no changes in oxidative stress and kidney function [72]. Similarly, in another double blind and placebo controlled resistance-training study, no decrease in visceral adipose tissue was observed; however a significant reduction in the cross-sectional area of visceral adipose tissue was noticed in the placebo group [22]. In this study, 30 overweight and moderately obese, but otherwise healthy middle-aged (35 to 55 yr) male subjects received 3.2 g/d CLA for 4 wk [22]. Pinkoski et al. [48] showed that supplementation of CLA (5 g/d) or placebo for 7 wk while resistance training (3 d/wk), which resulted in relatively lesser changes in body composition, accompanied by a lessening of the catabolic effect of training on muscle protein. Thus, some of the studies showed the effectiveness of CLA in fat mass reduction in subjects during resistance-training program. Contrary to this, no CLA-specific effects were observed on body composition, energy expenditure or appetite in non-obese, regularly exercising individuals (comprising 25 men and 27 women), who received either 3.9 g/d CLA or 3.9 g/d oleic acid rich sunflower oil (placebo) for 12 wk [51]. More recently, it was observed that CLA supplementation at a dosage of 6 g/d increased the level of total testosterone in the blood of human males, but no significant change was observed before or after each resistance exercise bout [73]. It suggests that CLA supplementation may promote testosterone synthesis through a molecular pathway that should be investigated in detail. Furthermore, this study becomes much relevant, since the correlation between the production of testosterone and body building still remains a controversy.

### CLA on fat-mass regain

Some pre-clinical studies showed that 10-CLA reduces fat uptake into adipocytes by lowering the activities of lipoprotein lipase and Δ^9^-desaturase, instead of enhancing lipolysis [14, 74, 75]. Based upon this background, a few clinical investigations were made on the effect of CLA on fat-mass regain after weight loss - with an assumption that CLA could block body fat gain. To check this, overweight adults were administered a very low-calorie diet for 3 wk, followed by CLA supplementation at a dosage of either 1.8 or 3.6 g/d for an intervention period of 13 wk [37]. Subjects took CLA in either dose showed increased regain of fat-free mass and resting metabolic rate, thereby lowering the regain of body fat relative to the control subjects. Interestingly, they concluded in later findings that the measures of appetite (hunger, satiety and fullness) favorably and dose-independently affected by the same dose of CLA but had no effect on energy intake at breakfast or improved body-weight maintenance after weight loss [76].

### CLA as dairy products

Apart from a few studies that investigated the effects of CLA supplementation in humans, there were some experiments designed to supplement CLA-enriched dairy products. Consumption of dairy products such as ultra-heat treated milk, butter, and cheese enriched with 1.42 g of 9-CLA did not significantly affect BFM and bodyweight in healthy, middle-aged men [77]. Another experiment compared the effects of the consumption of a modified butter, naturally enriched with CLA (4.22 g /100 g butter fat) on body composition in overweight and obese men, wherein abdominal adipose tissue area was measured by computed tomography [78], which found no differences in the accumulation of abdominal or subcutaneous adipose tissue, compared to the control group who consumed butter fat with low CLA (0.38 g /100 g butter fat) content. Consumption of a drinkable dairy product containing up to 3 g of 10-CLA isomer for 18 wk did not result in any significant effect on body composition in overweight, middle-aged men and women [46].

Venkatramanan et al. [45] examined the role of CLA enriched (so as to get 1.3 g/d) milk (*i.e.*, naturally enriched with only 9-CLA or synthetically with a mixture of 9- and 10-CLA isomers) in modulating body composition of moderately overweight, borderline hyperlipidemic individuals. More precisely, consumption of CLA-enriched milks (in either form) failed to alter TAG concentrations in the blood; body weight or fat composition [51]. Supplementation (14 wk) of 9- and 10-CLA isomers (in equal proportion, 70% purity) as TAG form blended in flavored yoghurt-like products was also found unable to alter body composition, although a significant increase in the resting metabolic rate was induced [53]. In contrast, a study conducted in Spain involving 60 healthy men and women (aged 35–65 yr) with signs of metabolic syndrome (BMI, 25–35 kg/m^2^) showed significant reduction (2-3%) of fat mass in overweight, but not in obese subjects; upon daily intake of 500 ml milk supplemented with 9- and 10-CLA mixture (3 g/d) for 12 wk [47]. Thus, in general, dairy products enriched with either of 9- or 10-CLA isomers or its mixture failed to establish a consistent effect on body composition.

### Long-term consumption of CLA

The question of inconclusive results on efficacy and effectiveness of CLA on body composition and obesity may answer from long-term intervention studies. Effects of any dietary supplement or food ingredient on body composition should be assessed over an extended period of time to conclude the results, because crash diet procedures seem inappropriate. In most of the studies, the intervention period lasted only for a few wk, and long-term studies were very few. In a study, 134 subjects including men and women were supplemented with 3.4 g CLA/d in the TAG or free FA form for 12 month, and extension study was also conducted in the same subjects for next 12 month [79]. During the first 12 month, significant reduction in BFM and leptin levels was reported. These changes in body composition were not related to diet and exercise. Most of the effects on BFM were observed during the first 6 month of CLA supplementation and the extension study concluded that CLA may be beneficial in preventing weight regain and long-term maintenance of BFM and LBM. These studies seem to be important as most of weight loss studies in overweight and obese subjects have demonstrated that most subjects will regain the lost weight within the next 1 to 2 yr [43, 80]. Gaullier et al. [66] also reported similar effects in another study with CLA supplementation for 1 yr in healthy overweight adults. Energy expenditure, substrate utilization and dietary fat oxidation were measured before and after 6 month of CLA supplementation, which showed that fat oxidation and energy expenditure increased during sleep in subjects received CLA, in comparison to placebo [81]. Supplementation of CLA (6.4 g/d) for 36 wk) reduced BMI and total adipose fat mass without altering LBM in obese postmenopausal women with type 2 diabetes, who were not also on a weight-loss diet or exercise plan [20]. Such long-term studies have to be conducted in a cross-over design (by including men and women of different age groups) to generalize the beneficial effects of CLA.

From these evidences, a few clinical studies pertaining to the long/short term effects of CLA in obese, sedentary, healthy or exercising humans have shown some beneficial effects of CLA in reducing body fat and improving body composition. However, all of them failed to reproduce the dramatic results reported in animal and *in vitro* models, especially mice. The extensive controversies in clinical studies limit from proposing a definite statement regarding the beneficial effects of CLA on body composition, so as to address the increasing concerns of health professionals, body builders and athletes.

### CLA and cardiovascular health

Hyper-triacylglycerolemia and elevated plasma cholesterol are suggested as the major risk factors for atherosclerosis and cardio-vascular diseases (CVD), and that blood lipid profile, blood pressure, BMI and blood sugar are generally considered as the indicators of heart health. The lipid profile is a panel of blood tests performed on the patient to determine the risk of CVD. These tests are good indicators of whether someone is likely to have a heart attack or stroke caused by blockage of blood vessels or hardening of the arteries (atherosclerosis). The lipid profile typically includes the baseline measurements of total cholesterol; high density lipoprotein cholesterol (HDL-C), often called good cholesterol; low density lipoprotein cholesterol (LDL-C), called bad cholesterol; and TAG in plasma [82]. Normal cholesterol levels vary by age and sex. LDL-C is the major cholesterol carrier in the blood, and if too much it is in circulation, it can slowly build up in the walls of the arteries of heart and brain leading to arteriosclerotic vascular diseases. According to American Heart Association, a high TAG level combined with low HDL-C or high LDL-C increases the risk of CVD [83]. The major circulatory markers associated with heart health are C-reactive protein (CRP), tumor necrosis factor-*α* (TNF-*α*), 15-*keto*-dihydro prostaglandinF2 (PGF2), 8-*iso-*prostaglandinF2-*α* (PGF2α), leptin, interleukin **(**IL)-6, plasma alanine transaminase, and total bilirubin [45]. Variations in the concentration of these markers in blood plasma (from the normal level) indicate dysfunctions of human system. The major circulatory markers associated with heart health and their normal levels in blood are listed in Table 3.

**Table 3:** **Major parameters of blood profile analysis, their normal level and variations in blood in relation to heart health ↑- increased; ↓- decreased**

Type	Normal	Indication
Total cholesterol	Below 200 mg/dL	↑ risk of heart disease
LDL-C	100-129 mg/dL	↓ risk of heart disease
HDL-C	40-50 mg/dL (men)	↑ protective against heart diseases
	50-60 mg/dL (women)	
Triglyceride	100-150 mg/dL	↑ risk of heart disease
VLDL-C	2-30 mg/dL	↑ risk of coronary artery disease
C-reactive protein	0-10 mg/L	↑ Inflammation/heart diseases
15-*keto*-dihydro PGF2	3-12 ng/L	↑ inflammation
8-*iso*-PGF2-*α*	150 ng/L	↑ oxidative stress
Tumor necrosis factor-α	extremely low/undetectable	↑ inflammation
Leptin	1-5 ng/dL (men)	↑ inflammation
	7-13 ng/dL (women)	

Some animal studies suggest the health benefits (anti-CVD effects) of CLA such as anti-sclerotic and improvements in blood lipid profile, hypolipidaemic and anti-oxidative effects [84–86]. Two different isomers of CLA (*i.e*., 9- and 10-CLA) have different or opposing effects on atherosclerosis [8]. The 10-CLA is pro-atherogenic and induces pathways involved in the development of insulin resistance, whereas 9-CLA is associated with reduced risk of CVD [87–89].

### Effect of CLA on blood lipid profile

Epidemiological studies showed that plasma HDL-C concentrations have an inverse relationship with the risk of CVD, and it is anticipated that raising plasma HDL-C levels might protect against atherosclerosis [83]. Supposed effects of CLA supplementation on blood lipid profile also remains inconclusive. Supplementation of an isomeric blend of CLA (9- and 10-CLAs in 50:50 ratio) for 12 wk (1.7 to 6.8 g/d) decreased total cholesterol, HDL-C and LDL-C [36]. Similarly, a dose of 0.7 or 1.4 g/d CLA decreased serum HDL-C and TAG significantly and increased the CLA content of serum lipids [25]. In this study, 22 volunteers were enrolled and they were divided into study and control groups in a doubly blind design; the study group received 0.7 g of CLA for four wk and 1.4 g of CLA for the next four wk, while the control group received similar dose placebo throughout. Diet was controlled, and no significant differences in energy or macronutrient intake were found between the two groups. A significant reduction of HDL-C was observed when 6.4 g/d CLA was consumed, whereas no change was observed in total cholesterol or LDL-C. But 2.1 g/d of CLA (9- and 10 CLAs in equal proportion) for 45 d showed no significant difference in serum TAG, total cholesterol, HDL-C in healthy non-obese sedentary women [52]. In contrast, CLA (9- and 10-CLA in 50:50 ratio) in a dose of 3.0 g/d for 8 wk increased total HDL-C concentrations by 8%. And the ratio of LDL-C to HDL-C was significantly reduced in subjects with stable, diet-controlled type 2 diabetes [90].

Supplementation (3 g/d) of a 50:50 isomeric blend of 9- and 10–CLAs or 80:20 blend of 9- and 10-CLAs for 8 wk showed that the former combination significantly reduced plasma TAG concentrations in synergy, whereas the latter blend significantly reduced the VLDL-C [91]. In this non cross-over double-blind, placebo-controlled and randomized study, 51 normolipidaemic subjects were enrolled. These results further suggested that CLA supplementation significantly improved the lipid profile in human subjects without any adverse effects on body weight, plasma glucose and insulin concentrations; and thus indicates the supposed cardio-protective effects of CLA. Contrary to this, the opposing effects 9- and 10-CLAs were observed by Tricon et al. [92]. In this cross-over study, 49 healthy men (20–47 yr; BMI 18–34 kg/m^2^) were enrolled, and supplemented with either 79.3% (2.38 g/d of 9-CLA) 9-CLA or 84.1% (2.52 g/d of 10-CLA) 10-CLA for 8 wk consecutively. It showed that 10-CLAincreased the ratios of LDL-C to HDL-C and total to HDL-C, whereas 9*-*CLA decreased them, suggesting the beneficial effects of CLA on blood lipid profile [93]. But, later, the same group showed that dairy products enriched with 9-CLA (1.42 g/d) had no significant effect on blood lipid profile [77].

Some studies observed neither a beneficial nor an adverse effect of an isomeric blend of 9- and 10-CLA in a ratio other than 50:50. A 93 d long study in 17 healthy female volunteers to observe the effect of dietary CLA (on blood lipids, lipoproteins, and tissue FA composition) showed that daily supplementation of 3.9 g/d CLA (the mixture contained 11.4% 9-CLA, 14.7% 10-CLA and 38.9% other CLAs) did not alter the blood cholesterol or lipoprotein levels of healthy, normo-lipidemic subjects [23]. Furthermore, no adverse effect of CLA supplementation was reported in this study, though plasma concentration of CLA was increased during the intervention period, *i.e*., 95.77% of the total CLA consumed was metabolized in the body [23]. In a cross-over study (for 6 month, n = 401, aged 40–70 yr and with a BMI > or = 25), consumption of 4 g CLA/d (4:1 ratio of 9- and 10-CLAs) did not influence aortic pulse wave velocity (marker of atherosclerosis), blood pressure, anthropometric characteristics, and concentrations of fasting lipid, glucose, insulin, and C-reactive protein (CRP), briefly it neither supported an anti-atherosclerotic effect nor an effect on CVD risk factors of 9- CLA [94].

Some studies investigated the effect of dairy products on lipid profile. However, human studies with the supplementation of CLA-enriched dairy products (*in situ* enrichment) produced contradictory results. Intake of 1.3 g/d of CLA in the form of naturally enriched milk (containing only 9-CLA) or milk enriched with a synthetic mixture of 9- and 10-CLAs for 8 wk did not alter the levels of cholesterol, LDL-C, HDL-C or TAG concentrations in blood samples of moderately overweight, borderline hyperlipidemic individuals [45]; likewise, in another cross-over study (contained healthy middle-aged men, *n* = 32 for 6 wk), supplementation of dairy products (heat-treated milk, butter or cheese) enriched with 9*-*CLA and VA (1.421 g/d) appeared to have no effect on the blood lipid profile of men [77]. However, levels of CLA and VA in human milk can be modulated if breastfeeding mothers replace conventional dairy and meat products with organic dairy products (enriched by natural feeding) [95]. Recently, Penedo et al. [96] showed that intake of butter, naturally enriched with 9-CLA (1.02 ± 0.167 g/d) for 8 wk induced beneficial changes in immune modulators associated with sub-clinical inflammation in overweight individuals. Furthermore, sheep cheese naturally enriched in VA, CLA and ALA improved the lipid profile and reduced anandamide (an endogenous cannabinoid neurotransmitter and obesity marker) in adults with diagnosed mildly hypercholesterolaemia [97].

### CLA on circulatory markers

CRP is synthesized by liver in response to inflammation. Inflammations may be due to a variety of reasons such as cancer, diabetes, cardiovascular diseases, *etc*. [98, 99]. Normal concentration of CRP in healthy human serum is usually lower than 10 mg/l, but slightly increasing with aging. Increased plasma concentration of CRP, a circulatory inflammation marker helps in predicting CVD [100]. Supplementation with10-CLA for 12 wk markedly increased the levels of PGF_2_α (578%) and CRP (110%), compared with placebo in 60 men with metabolic syndrome [60]. A dose of 3 g 9-CLA/d significantly elevated the levels of urinary PGF2 and PGF_2_α - the markers of *in vivo* inflammation and oxidative stress, respectively; after the supplementation for 3 month with 25 abdominally obese men against olive oil as placebo [87]. A mixture of 9- and 10-CLA isomers with equal proportions also reported an increased CRP, but not of the other inflammatory markers, *i.e*., TNF-α, TNF-α receptors 1 and 2, and vascular cell adhesion molecule (VCAM)-1 [101]. Another study concluded that a mixture of 9- and 10-CLAs had more adverse effects on CVD markers, while 9-CLA isomer appeared to be more neutral in healthy postmenopausal women. Daily supplementation of 5.5 g of CLA mixture significantly elevated the level of CRP, fibrinogen, and plasminogen activator inhibitor-1 in plasma [102]. The CLA mixture at a dose of 3.0 g/d reduced fibrinogen concentrations but had no effect on other inflammatory markers of CVD like CRP and interleukins (IL) in subjects with type 2 diabetes [90]. High dose of CLA consumption (6.4 g/d of 9- and 10-CLA in 50:50 ratio) for 12 wk markedly increased the levels of CRP and IL-2, suggesting an increase in inflammation during short-term supplementation. In contrast, CLA in the same composition (50:50 ratio), but in lower dose (*i.e*., 3.0 g/d showed) no effect on the inflammatory markers of CVD (CRP and IL-6) [40].

Outcome from some recent studies suggested that CLA did not increase the risk of CVD. Pfeuffer et al. [44] assessed the effect of CLA against safflower oil on endothelial function and markers of CV risk in overweight and obese men, *i.e*., by the consumption of 4.5 g/d of the CLA isomeric mixture for 4 wk. It was observed that CLA did not impair endothelial function. Other parameters associated with metabolic syndrome and oxidative stress were not changed or slightly improved. Interestingly, it was observed that oral supplementation of CLA along with calcium reduced the incidence of pregnancy-induced hypertension without changing the plasma levels of other circulatory markers such as PGF2α, CRP and IL-6 [103]. Forty eight healthy primigravidas with a family history of preeclampsia and with diastolic notch were included in this double-blind and placebo-controlled non cross-over study. Participants were randomized to daily oral doses of elemental calcium (0.6 g/d) with CLA (0.45 g/d) or lactose-starch placebo from wk 18 to wk 22 of gestation until delivery.

**Figure 2:**
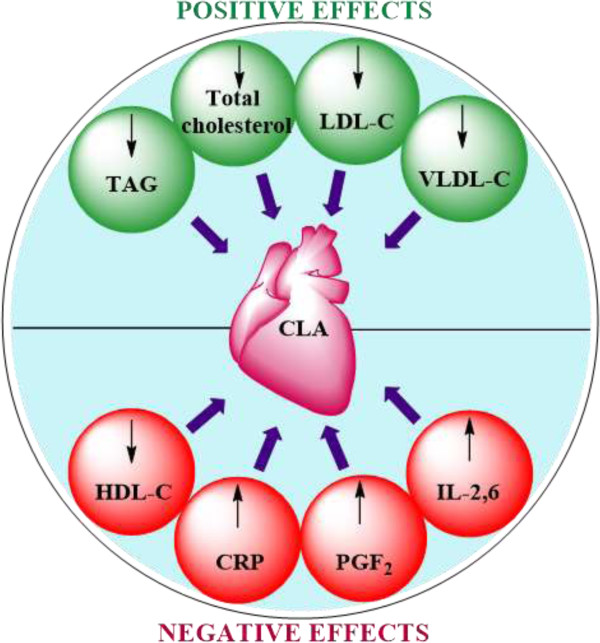
**Proposed effects of CLA consumption on heart health.**

The controversial beneficial and detrimental effects of CLA on heart health observed during clinical studied are summarized in Figure 2. All these studies were too randomized in dosage, composition and duration, which make difficult to conclude the positive effects of CLA on heart health. Moreover, there is a complete lack of uniformity in assessing the effects CLA on heart health, *i.e*., some studies were focused on lipid profiles, while others on circulatory markers; but none of them was reported to have a consistent effect. Even though the isomeric mixture of 9- and 10 CLA (1:1) was found to exert some positive effects, it is necessary to elucidate the mechanism of action to ascertain which of these isomers elicited the effect.

### CLA and immune function

Different studies show that the effects of dietary CLA on immune functions in animal as well as human models are highly variable and inconsistent (Table 4). For instance, a 93 d long study in 17 young women upon feeding with 3.9 g/d CLA isomeric blend showed no alteration in any of the indices of immune status such as circulating white blood cells, granulocytes, monocytes and lymphocytes [104]. Even after immunization with influenza vaccine, the delayed type hypersensitivity response and serum antibody titers were not altered during the intervention period. These data suggest that short-term CLA supplementation in healthy volunteers was safe, but it showed no added benefit to their immune status [104]. Moreover, short-term consumption of CLA produced no observable physiological change in blood coagulation and platelet function in healthy adult females [23]. CLA supplementation (3.9 g/d of a mixture of CLA isomers: 17.6% 9-CLA, 10-CLA, 23.6% *cis*-11,*trans*-13 CLA, 16.6% *trans*-8,*cis*-10 CLA and other isomers 19.6%) resulted in an eight folds increase (0.12 to 0.97, wt %) in the concentration of CLA in the lipid fraction of peripheral blood mononuclear cells (PBMC) without changing the concentration of other FAs; but, increased concentration of CLA did not alter the functions of PBMCs, *i.e*., secretion of PGE2, leukotriene B4, IL-1β or TNFα [105]. Supplementation with 9- to10 CLA in the ratio 50:50 or 80:20, respectively resulted in 35% increased CLA levels in PBMC [106]. Interestingly, in this non cross-over study, 62% of the subjects, who consumed 9- and 10-CLA mixture in the ratio 50:50 showed increased titers of protective antibody levels after hepatitis B vaccination. Although the overall effect was not significant, the results at least suggested that 50:50 CLA might have a biologically relevant enhancing effect on the response to hepatitis B vaccination, which warrants further study [106]. Contrary to this, supplementation with the 9- and 10-CLA isomers (80:20 blend, respectively) significantly enhanced phyto-hemagglutinin (PHA) content, a T-cell mitogen-induced lymphocyte proliferator. CLA decreased basal IL-2 secretion, but increased PHA-induced IL-2 and TNF-α production - when 55 healthy volunteers received 3 g/d of 9- and 10-CLAs blend in ratios 50:50 and 80:20, respectively [107]. Plasma IgA and IgM levels were found increased upon supplementation with 9- and 10-CLAs (50:50), but decreased the levels of IgE, TNF-α and IL-1β. In addition to these effects, delayed hypersensitivity response was decreased during CLA supplementation [108].

**Table 4:** **Major clinical trials investigating the effect of CLA consumption on immune status; ↑- increased; ↓- decreased; ↔ no change in**

Subjects	Dose	Duration	Observation	Reference
17 women	3.9 g CLA (Tonalin)/d	93 d	↔ immune status	[104]
17 women	3.9 g CLA (Tonalin)/d	93 d	↑ PBMC	[105]
			↔ circulatory cytokines	
71 males	1.7 g 9 & 10-CLA (50:50), Clarinol™/d	12 wk	↑ protective antibodies upon vaccination for hepatitis B	[106]
49 healthy men	2.38 g/d 9-CLA or	8 wk	↓ mitogen-induced T-lymphocyte activation	[93]
	2.52 g/d 10-CLA		↔ circulatory cytokines	
55 healthy volunteers	2 g 9 & 10-CLA (50:50)/d or	8 wk	↔ markers of human immune function	[107]
	1.76 g 9 & 10-CLA (80:20)/d			
28 men and women	3 g 9 & 10-CLA (50:50)/d	12 wk	↑ levels of IgA, IgM and IL-10	[108]
			↓ TNF-α, IL-1β and delayed type hypersensitivity response	
28 mild asthmatic adults	4.5 g CLA/d	12 wk	↑ airway hyperresponsiveness	[109]

### CLA *vs*. asthma

CLA is reported to modify the inflammatory responses associated with allergic airway disease, primarily in animal models. A prominent study in this regard came from the group of MacRedmond et al. [109], which demonstrated that supplementation of 4.5 g/d CLA as an adjunct to usual care in overweight mild asthmatics (28 subjects; aged 19–40 yr involved) for 12 wk was well tolerated, which was associated with improvements in airway hyper-responsiveness [109]. However, daily supplementation of 4.8 g CLA for 8 wk did not attenuate airway inflammation or hyperpnea-induced broncho constriction in asthmatic individuals [110].One of the early studies in this direction measured the mean serum phospholipid esterified 9-CLA concentration in peripheral blood; observed it as significantly higher in 98 patients with chronic stable asthma, and 25 patients with acute severe asthma. Thus the supposed role of oxygen-derived free-radical activity in inflamed lung tissue was envisaged [111]. It shows that, some attempts were made to estimate the effect of CLA on immunity with reference to asthma, but none of them succeeded in reproducing the positive effects such as enhancement of immune function, down regulation of autoimmunity and increased proliferation of lymphocytes*,* consistently in clinical studies [112–114]. Furthermore, activation of peroxisome proliferator-activated receptors (PPARs, a group of nuclear receptors), especially PPAR-*γ* in the human airway smooth muscle would be a possible strategy to treat airway diseases [115]; therefore, targeting PPAR-*γ*, 9-CLA might show therapeutic value in alleviating airway disease by affecting epithelial and eosinophil functions [116].

### CLA and cancer

The interest on CLA mainly sprouted from the discovery of the anticancer property of CLAs [11]. Nevertheless, only a few studies have examined the isomers-specific effects of CLA in humans. In fact, no clinical studies have been conducted to relate CLA consumption with the incidence of cancer, but the data available in this regard are only from epidemiological studies. Such data can be viewed as a collection of statistical tools used to elucidate the associations of CLA exposures to health outcomes. Regarding clinical studies on cancer, many researchers focused on human breast cancer; for instance, in an elaborate follow-up study using Cox proportional hazards models; Larsson et al. [117] showed that the dietary intake of CLA had no evidence for a protective role against breast cancer development in women. Chajes et al. [118] conducted a case–control study among 297 women treated for breast cancer or benign breast disease at the University Hospital of Tours, France, to evaluate the hypothesis that CLA protects against breast cancer, and they could not show a link for the negative association between adipose tissue CLA (predominantly 9-CLA) and the risk of breast cancer.

High-fat dairy food and CLA intake were examined in 60,708 women of age 40 to 76 (Swedish mammography cohort study) with 14.8 yr follow-up. It was found that women who consumed four or more servings of high-fat dairy foods per day (including whole milk, full-fat cultured milk, cheese, cream, sour cream and butter) showed half the risk of developing colorectal cancer, compared to women who consumed less than one serving per day [119]. Concerning CLA intake, they found it was associated with an almost 30 percent reduction in the risk of colorectal cancer [119]. Similarly, the possible role of CLA in preventing testicular cancer was depicted by the decreased CLA content in mitochondrial fractions of testicular cancer as against the normal testicular cells; and that CLA incorporation into nuclei and cytosol was significantly higher than its incorporation into plasma membranes and mitochondria [120]. Tumors in estrogen receptor (ER)-negative epithelial cells in the breast are common among premenopausal women [121]. McCann et al. [121] demonstrated that the protective effect of 9-CLA in women with its higher intake, *i.e*., the number of ER-negative cells to ER-positive was found decreased in such women. Another epidemiological study (the Netherlands cohort) with 6.4 yr of follow-up evaluated the relation between intakes of CLA and other FAs failed to confirm the anti-carcinogenic property of CLA in humans with breast cancer incidence [122].

A few studies examined the relationship between dietary or serum CLA in women and the risk of breast cancer. Such studies found an inverse association between dietary and serum CLA and risk of breast cancer in postmenopausal women [123]. But in contrast, the adipose tissue extracts from a population of French patients with invasive breast carcinoma failed to reveal any positive correlation between adipose tissue CLA and the incidence of breast cancer [124]. Since CLA accumulates in body fat stores, the adipose tissue of breast cancer obtained at the time of surgery could be used as a qualitative biomarker for CLA intake.

Thus, the available human clinical studies could not ascertain the anti-cancer property of CLA. A major limitation in the epidemiological studies is the difficulty in obtaining accurate estimates of dietary CLA intake. Most of the studies were carried out in small populations, where the diversity in food habits was less. Moreover, no clinical studies evaluated the effects of pure CLA preparations or individual isomers on the incidences of cancer. It focuses that well-defined and controlled studies are required to fully understand the effects of CLA intake on the incidence of human cancer.

### CLA and diabetes

The life style epidemics, diabetes and obesity are considered as the major causes of morbidity and mortality all over the world; and that obesity and weight gain are associated with an increased risk of diabetes [125]. The hormone, insulin is responsible for regulating glucose concentration in blood. Insulin resistance is a state in which cells do not respond properly to insulin (even if it is available in the blood), which leads to hyperinsulinemia (high blood insulin). Some animal studies demonstrated that CLA supplementation enhances insulin sensitivity; however, the mechanism underlying this effect is unclear [126, 127].

Relatively few studies have examined the anti-diabetic properties of CLA in humans. Supplementation of 3.0 g/d of CLA (in 24 women) for 64 d showed no significant changes in the levels of circulatory glucose or insulin [128]. CLA isomeric blends at the same dose also showed no significant effects on plasma glucose or insulin levels in healthy human subjects [91]; in such studies, fasting blood glucose and/or insulin generally showed little demonstrable effect. The gold-standard for quantifying blood glucose is the ‘hyperinsulinemic-euglycemic clamp test’, which measures the amount of glucose necessary to compensate for an increased insulin level without causing hypoglycemia [129]. However, another study utilizing a euglycemic/hyperinsulinemic clamp in abdominally obese male subjects indicated a decline in insulin sensitivity after supplementation with both mixed and purified 9- and 10*-*CLA isomers at a dose of 3.4 g/d for 12 wk [65]. The 10-CLA supplementation increases oxidative stress and inflammatory biomarkers in obese men [60]. Oxidative stress seems closely related to induced insulin resistance, which suggests a link between the FA-induced lipid peroxidation; these unfavorable effects of 10-CLA might be of clinical relevance with regard to CVD [60]. Recently, Shadman et al. [50] showed that supplementation of CLA (50:50 isomer blend of 9- and 10-CLA) with or without vitamin E for 8 wk showed a trend to increase in malondialdehyde (a marker of lipid peroxidation) in overweight type 2 diabetic patients.

In non-diabetic abdominally obese men, 3.4 g/d 10-CLA supplementation for 12 wk induced hyperproinsulinaemia (plasma proinsulin, insulin, C-peptide and adiponectin concentrations, including their associations with change in insulin sensitivity assessed), which was related to impaired insulin sensitivity, independently of changes in insulin concentrations [130]. These results are of clinical interest, as hyperproinsulinaemia predicts diabetes and cardiovascular diseases. The same investigators also showed that the other active isomer of CLA (9-CLA) also increased insulin resistance in abdominally obese individuals upon its supplementation at a dose of 3 g/d for 3 month. [87]. But, the isomeric mixture of 9- and 10-CLA (3.4 g/d for 6 month) showed no significant effect on glucose metabolism or insulin levels in overweight or obese individuals [131]. All these studies failed to support the anti-diabetic property of CLA in humans; however administration of 4 g/d of mixed CLA isomers improved insulin sensitivity in young sedentary humans [132]. Sixteen individuals (age, 21.5 ± 0.4 yr; body mass, 77.6 ± 3.4 kg) were involved in this study. Ten subjects received 4 g/d of mixed CLA isomers (35.5% 9-CLA; 36.8%10-CLA) for 8 wk, whereas six subjects received placebo (safflower oil); but this study was conducted in a group of 16 subjects - too small to offer a generalized effect .

Clinical studies regarding the anti-diabetic effects of CLA are inconclusive. Rather, some of them speculated the reduction in insulin sensitivity; which attract immediate attention of the medical practitioners, because the increased consumption of CLA through dietary supplements might be ill-advised.

### Adverse effects of CLA consumption

It seems that the use of weight-loss supplements containing 9-CLA, 10-CLA or both as mixture is worrying, because most of the clinical studies presented in the previous sections provide mostly neutral or inconclusive results with very few favorable impacts (Table 5). In association with this, a few studies reported some adverse effects such as oxidative stress, insulin resistance, gastrointestinal irritation, *etc*., but no serious adverse effects were reported at the time of intervention except the relapse of asthma on consumption of 3.4 g/d of CLA [36]. Therefore, most of these side effects could be categorized as ‘mild to moderate’.

**Table 5:** **Proposed beneficiary and detrimental effects of CLA from clinical studies**

Diseases	Positive effects	Negative effects
Obesity	Reduced body fat mass	Oxidative stress
	Reduced body mass index	Abdominal irritations
	Reduced body fat percentage	
	Reduced body fat regain	
	Increased lean body mass	
	Improved muscle mass	
Cardiovascular diseases	Improved blood lipid profile	Enhanced production of circulatory markers of oxidative stress
	Reduced total cholesterol	
Immune disorders	Enhanced the levels of protective antibodies	Elevated levels of inflammatory markers in circulation
	Induced lymphocyte proliferation	
	Reduced delayed hypersensitivity responses	
Cancer	Reduced the risks of colorectal, testicular and breast cancers	Increased oxidative stress
Diabetes	Enhanced insulin sensitivity	Dysregulation of blood glucose and insulin
		Insulin resistance
		Decreased expression of GLUT4

### Oxidative stress

Many studies showed increase in the plasma concentration of CLA, which was directly proportional to the quantity of CLA consumed [23, 133]. Therefore, the immediate expected biological effect is oxidative stress. Oxidative stress is the reflection of an imbalance between the systemic manifestation of reactive oxygen species and the ability of the body system to readily detoxify them or to repair the resulting damage imparted to cell components like proteins, lipids and nucleic acids. It is known that the free radicals such as reactive oxygen formed by lipid peroxidation would ‘steal’ electrons from the lipids in cell membranes, resulting in cell damage [134]. Prolonged oxidative stress may lead to cancer and heart diseases [134]. Supplementation with 10-CLA dramatically increased the rates of oxidative stress, to the levels considerably higher than that observed in heavy smokers [60]; it also enhanced the release of inflammatory biomarkers in obese men [60]. Long-term CLA supplementation studies lasting for one and two years have found to be well tolerated, but there was an increase in circulatory markers of inflammation such as CRP, TNFs, and ILs [59, 102]. Changes in these markers of inflammation and oxidative stress could be related to the increase in insulin resistance associated with the risk of cardiovascular disease [79, 135]. Administration of CLA (4.2 g/d) for three month significantly induced both non-enzymatic and enzymatic lipid peroxidation, which was suggested to cause cell damage [136].

### Insulin resistance

Insulin resistance is a physiological disorder, under which the cells fail to respond to the normal actions of the hormone insulin, though it is sufficiently produced by the body – this impairment leads to hyperglycemia (*i.e*., type 2 diabetes). Decreased sensitivity or resistance towards insulin upon consumption of CLA was observed in some studies [60, 65]. Riserus et al. [65] showed with obese men that10-CLA might modulate insulin resistance in humans, and that oxidative stress is closely related to induced insulin resistance, as evidenced from the increased levels of the marker, 8*-*iso-prostaglandin-F_*2α*_ in plasma. Furthermore, insulin resistance is closely related to the impairment (decrease) of the expression of glucose transporter-4 (GLUT4), a membrane transporter of glucose. It was proven beyond doubt that 10-CLA decreases the expression of GLUT4 [137], which shows that indiscriminate use of 10-CLA to treat obesity would lead to type 2 diabetes as the immediate side effect, this would further damage blood vessels and thereby increased risk of CVD [138]. Moreover, unutilized insulin (due to resistance) in plasma can contribute to increased appetite (especially for carbohydrates and sugary foods), which would add to the gravity of CVD.

### Irritation of intestinal tract

A few studies showed mild irritations of intestinal tract such as irritation [60], laxative effects and flatulence [47], gas bloating [20], indigestion, diarrhea and nausea [36, 48] in subjects consumed CLA. Most of these effects were considered as mild to moderate and were transient; and one may assume that these effects may be due to the capsule material or the oily nature of the substance or initial adaptive problem with the lipid nutrient.

### Milk fat depression

Consumption of commercial CLA reduced the fat content in cows [139]. Since milk is the only source of nutrients for infants, decreased milk fat in lactating humans is another concern regarding the CLA consumption. Masters et al. [140] showed that CLA consumption significantly reduced the milk fat without affecting the total milk output. However, another two human studies found no changes in milk fat or protein [141, 142], but in these studies, the intervention period was too short (about a wk) to arrive at a conclusive result.

### Cross-talk on CLA consumption

General view on CLAs is that the 10*-*CLA exerts specific effects on adipocytes and liver, whereas both the 9- and 10-CLAs appear to be active in inhibiting carcinogenesis [14]. It is likely that the inconsistent and often contradictory results on the effectiveness of CLA consumption in human health could be the outcome of a number of factors, including differences in subject groups, age, quantity and duration of CLA intake, composition of CLA mixture, purity of CLA, acceptance of the CLA by the body, food intake, gender and racial differences, genetic polymorphism and also the executed measurements (parameters studied) for assessing the effect. Moreover, crucial factors that impact research outcomes include the nature of control supplement (placebo), and study design (cross-over *vs*. non cross-over designs), because, the efficacy of CLA supplements remains inconsistent in cross-over and non cross-over randomized clinical studies [143].

### Plasma content of CLA

Determination of a normal CLA content in the blood plasma could help in estimating if a person consumes satisfactory amounts of CLA with the diet, and thus takes advantage of its potential beneficial effects on health. The only CLA isomer that appears in higher percentage than the detection limit (0.03% of total FAs) is 9-CLA [133]. They arrived at this conclusion based on the data obtained from 3 groups of individuals (n = 12 for each group), *i.e*., who not consumed dairy products, individuals consumed normal amounts of dairy products (about 50 g/d cheese) and individuals consumed 1.4 g CLA/d as supplement (both 9- and 10-CLAs in equal proportion). The duration of this study was 6 months, and in the last group who consumed CLA supplement, the average CLA content in plasma was 0.2% of the total FAs with no untoward side effects. The blood samples were collected for analysis in the morning (in the fasted state) after a 12 h restriction for meal and drinks. Thus, individuals who have 9-CLA levels in their blood plasma within the range up to 0.09% of total FAs could serve as ideal participants in future CLA supplementation studies.

### CLA supplements *vs.*placebo

In most of the clinical studies, vegetable oils such as sunflower oil, olive oil, safflower oil and soybean oil (Table 2) have been used as placebo in the form of capsules or pills [49, 66, 90]. In fact, the proportion of MUFA and PUFA, especially LA present in these placebo oils (for instance, the predominantly used sunflower and olive oils) are not properly addressed by the researchers. According to WHO (Codex International food standards), sunflower oil, soybean oil, olive oil and safflower oil contained significant levels of MUFA and PUFA, which include OA, LA and ALA (Table 6) [144, 145]. It was thought that VA is the only precursor of CLA in humans. However, non-ruminal bacteria inhabiting human gastro-intestinal (GI) tract like *Lactobacillus acidophilus* and *L. casei* isolated from intestine [146], *Bifidobacterium bifidum* and *B. breve* isolated from the fecal matter of neonates [147], and *Ruberia* spp. isolated from intestine [148] could efficiently produce 9-CLA from LA, probably though the mediation of VA (in tissues) or 10-hydroxy octadeceinoic acid (18:1) [148], as occurring in ruminal biohydorgenation. In addition to 9-CLA, *Lactobacillus* spp. also synthesizes 10-CLA and *trans*-9, *trans-*11-CLA [146]. From this, it is evident that a portion of LA in placebo oil would be biohydogenated by the bacteria residing in GI tract (as in rumen) into CLA through the mediation of VA. Irrespective of this fact, most of the clinical studies use the aforesaid vegetable oils as placebo, neglecting their effects on human health; especially their supposed supplementary and complementary effects. The dietary intake of the precursor VA was found to have some major effects on heart health, blood lipid profile and immunity, and also protective against fatal ischemic heart disease [149–151]. This would lead to the misinterpretation of the results, *i.e*., false-positive results. Therefore, during clinical studies, the composition of FA in placebo and its effects on human health need to be addressed with due respect, and independently for getting reliable results.

**Table 6:** **Various vegetable oils used as placebo in clinical studies with their polyunsaturated fatty acids (PUFA) content including LA**

Vegetable oil	Total PUFA*	LA*
Sunflower oil	66	66
Flax seed oil	66	13
Safflower oil	42	41
Olive oil	10	9
Soybean oil	58	51

*Units: grams fatty acids per 100 grams oil.

### Selection of the subjects

The clinical studies with CLA lack a common protocol for selecting the subjects. Description of the subjects including gender and age, medical treatments given prior to intervention are the critical factors to be considered while selecting the subjects. Medical history of the subjects should also be recorded before concluding the safety and efficacy concerns of CLA consumption. In most of the studies, the subjects selected were categorized and designated as normal, healthy obese, with metabolic syndrome, with insulin resistance, *etc*. This arbitrary classification for the convenience of the investigator poses a question *i.e*., which is the most suitable model to study the health effects of CLA? Is it with the designation normal, obese, immune-compromised subjects with metabolic syndrome or with other diseases?

Another factor to be considered in clinical studies is the continental, racial and gender differences among the subjects; for instance, literature shows that most of the clinical studies on CLA were performed in North America and Europe. The reproducibility of such results in racially and continently separated populations all over the world, especially in Asia, Africa and South America is another point of concern, which has to be verified before accepting the nutritional status of CLA in modulating biological functions.

### Dosage and duration

Other factors of concern are the composition, dosage and duration of CLA consumption. If not otherwise stated, composition and purity of CLA are meant for 9- and 10 CLAs. Generally, human studies use a CLA mixture (about 40:40) of 9- and 10-CLAs; and proportion of CLA isomers depends mainly on the nature of substrate, mode of synthesis (production), physico-chemical parameters involved in synthesis, and purification strategies adopted [152, 153]. Even if stated as purified, the purity would be about 80%; and the remaining 20% would be represented by other CLA isomers and unmodified FAs. These ‘impurities’ would also contribute to the inconclusive results, as different isomers proved to have different physiological actions in human body. Most of clinical studies evaluated the effects of CLA consumption for a short period, usually of 4–12 wk. But Gaullier et al. [66, 79] conducted comparatively long-term study spanning for 1–2 yr. Generally, in these studies, CLA (isomer or mixture) dosages varied from 0.7 g/d to 6.8 g/d per human and administered mostly in the form of TAGs or free FAs. The dosage of CLA administration in humans is also very low, compared to animal studies (in terms of body weight); thus the results in pre-clinical animal studies (high dose) may not be comparable with the real clinical studies. Therefore, CLA dose (intake) may be considered based on energy percentage. Two people with the same body weight may have a very different body composition (*e.g*., women *vs*. men; body builder *vs*. obese person), which in turn impacts the metabolism differently.

Another crucial question is the retention of the so-called good effects for a long time; of course, one might expect that CLA should be consumed as if drugs are taken for chronic diseases. Unlike in mechanistic *in vitro* studies, the criss-crossed signaling pathway through which CLA induce its effects has to be elucidated clearly in clinical studies. Moreover, the biological effects of individual CLA isomers, mainly 9- and 10-CLA, their synergistic interactions and even the possible opposition between the isomers have to be unveiled.

### CLA and adjuncts

Effect of CLA consumption along with various adjuncts is another area of clinical research that has to be studied evidently. Some studies showed the positive health benefits of CLA are related to heart health and body fat reduction on consumption along with calcium, VA, whey proteins and oryzanol [49, 68, 103]. CLA (6 g/d) supplementation along with creatine and whey protein resulted in enhanced strength improvements and LBM with high-volume strength training in well-trained young adults [72]. CLA consumption along with other PUFA was found to have protective effect against renal carcinoma [120]. Therefore, an effective combination of CLA along with other supplements or with *ω*-3 FAs has to be addressed to reveal the possible real effects of CLA consumption on human health.

## Conclusions

As far as the voluminous literature on CLA is concerned, only a few studies to date examined the effects of CLA in humans *in vivo*. However, results of these studies do not reflect the dramatic and consistent data demonstrated in animal studies. Thus, these disappointing results in humans demand more precise experimentations with humans. The interest in CLA research still persists, and hence, many questions related to the safety and efficacy on the consumption CLA have to be answered scientifically. Hence, it is imperative to critically evaluate and consolidate prominent findings on human consumption of CLA, *i.e*., the principal actions of this minor lipid nutrient exerting on human system so that future investigations would focus on specific CLA isomers and the most reasonable mechanism of action due to them. One of the major limitations in human studies is that most of the studies depend only on the blood cells or plasma, and fat deposition. Thus, majority of the clinical studies failed to provide conclusive evidences for the effectiveness of CLA on human health, except for anti-obesitic properties which offered a little hope to prevent body weight regain though fat deposition, nevertheless increased oxidative stress and insulin resistance due to such over-consumption of CLA poses contradictory concerns. Moreover, age, gender, genetic polymorphism and immune status of the subject, role of other nutrients present in the diet, and extend of absorption of individual isomers to different tissues have to be well addressed during the intervention period – so as to evaluate the safety and efficacy of CLA consumption on human health. As far as human consumption of CLA is concerned, a definite conclusion for safety and efficacy has not been reached yet. At this context, we strongly recommend the need for more precise and well-designed long-term intervention studies with controlled food intake and activity level to assess the effectiveness of CLA on human health. Moreover, such studies need to be duplicated in other laboratories giving emphasis to men and women, age group, ethnic background, food style, continental and even national uniqueness, cultural and geographic barriers, *etc.* without comparing data from animal studies – *i.e*., a real double-blind clinical study. *In toto*, clinical evidences indicate a possible link of supplemental CLA *per se* toward negative or inconclusive outcomes; thus, inclusion of CLA in the *Codex Alimentarius* (Book of Food) – which describes internationally recognized standards of food – may be considered.
